# Tetra­kis(quinolin-8-olato-κ^2^ *N*,*O*)hafnium(IV) toluene disolvate

**DOI:** 10.1107/S1600536809043244

**Published:** 2009-11-04

**Authors:** J. Augustinus Viljoen, Hendrik G. Visser, Andreas Roodt, Maryke Steyn

**Affiliations:** a Department of Chemistry, University of the Free State, PO Box 339, Bloemfontein 9300, South Africa

## Abstract

In the title compound, [Hf(C_9_H_6_NO)_4_]·2C_7_H_8_, the hafnium metal centre is coordinated by four *N*,*O*-donating bidentate quinolin-8-olate ligands arranged to give a square-anti­prismatic coordination polyhedron with a slightly distorted dodeca­hedral geometry. The average Hf—O and Hf—N distances are 2.096 (3) and 2.398 (3) Å, respectively, and the average O—Hf—N bite angle is 70.99 (11)°. The crystal packing is controlled by π–π inter­actions between quinoline ligands of neighbouring mol­ecules and hydrogen-bonding inter­actions. The inter­planar distances vary between 3.138 (1) and 3.208 (2) Å, while the centroid–centroid distances range from 3.576 (1) to 4.074 (1) Å.

## Related literature

For a Zr analogue of the title compound, see: Lewis & Fay (1974 ▶). For hafnium and zirconium *β*-diketonato complexes, see: Viljoen *et al.* (2008 ▶, 2009 ▶); Demakopoulos *et al.* (1995 ▶), Zherikova *et al.* (2005 ▶, 2006 ▶, 2008 ▶); Steyn *et al.* (2008 ▶); Calderazzo *et al.* (1998 ▶).
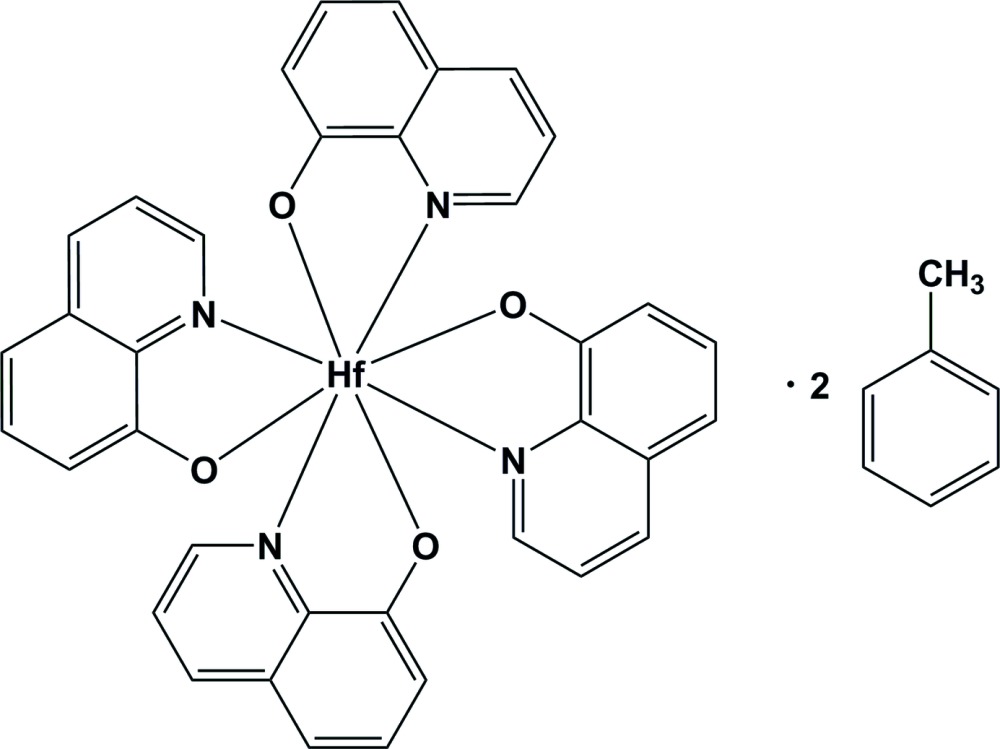


## Experimental

### 

#### Crystal data

[Hf(C_9_H_6_NO)_4_]·2C_7_H_8_
*M* *_r_* = 939.35
Triclinic, 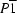

*a* = 11.3323 (5) Å
*b* = 12.5539 (5) Å
*c* = 15.7126 (7) Å
α = 69.746 (2)°
β = 69.700 (2)°
γ = 75.787 (2)°
*V* = 1946.79 (14) Å^3^
*Z* = 2
Mo *K*α radiation
μ = 2.73 mm^−1^
*T* = 100 K
0.22 × 0.10 × 0.04 mm

#### Data collection

Bruker X8 APEXII 4K Kappa CCD diffractometer
Absorption correction: multi-scan (*SADABS*; Bruker, 2004 ▶) *T*_min_ = 0.585, *T*_max_ = 0.899
22928 measured reflections
8458 independent reflections
7551 reflections with *I* > 2σ(*I*)
*R*_int_ = 0.044

#### Refinement

*R*[*F* ^2^ > 2σ(*F* ^2^)] = 0.033
*wR*(*F* ^2^) = 0.100
*S* = 1.04
8458 reflections
534 parameters
H-atom parameters constrained
Δρ_max_ = 1.16 e Å^−3^
Δρ_min_ = −0.81 e Å^−3^

### 

Data collection: *APEX2* (Bruker, 2005 ▶); cell refinement: *SAINT-Plus* (Bruker, 2004 ▶); data reduction: *SAINT-Plus*; program(s) used to solve structure: *SIR92* (Altomare *et al.*, 1999 ▶); program(s) used to refine structure: *SHELXL97* (Sheldrick, 2008 ▶); molecular graphics: *DIAMOND* (Brandenburg & Putz, 2005 ▶); software used to prepare material for publication: *WinGX* (Farrugia, 1999 ▶).

## Supplementary Material

Crystal structure: contains datablocks I, global. DOI: 10.1107/S1600536809043244/bg2301sup1.cif

Structure factors: contains datablocks I. DOI: 10.1107/S1600536809043244/bg2301Isup2.hkl

Additional supplementary materials:  crystallographic information; 3D view; checkCIF report

## Figures and Tables

**Table 1:** Hydrogen-bond geometry (Å, °)

*D*—H⋯*A*	*D*—H	H⋯*A*	*D*⋯*A*	*D*—H⋯*A*
C105—H105⋯O1^i^	0.93	2.56	3.467 (5)	166

Symmetry code: (i) 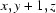.

**Fig. 1.**
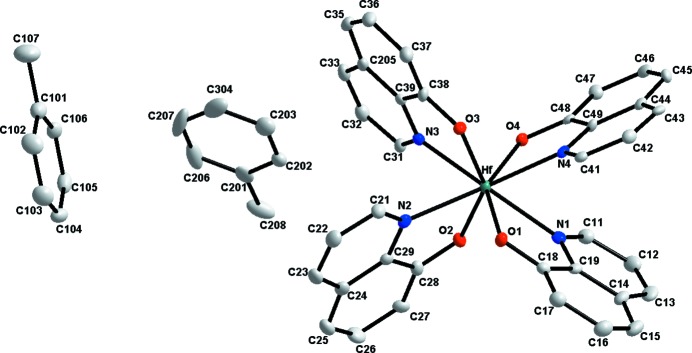
Representation of the title compound (I), showing the numbering scheme and displacement ellipsoids (50% probability). H atoms omitted for clarity.

**Fig. 2.**
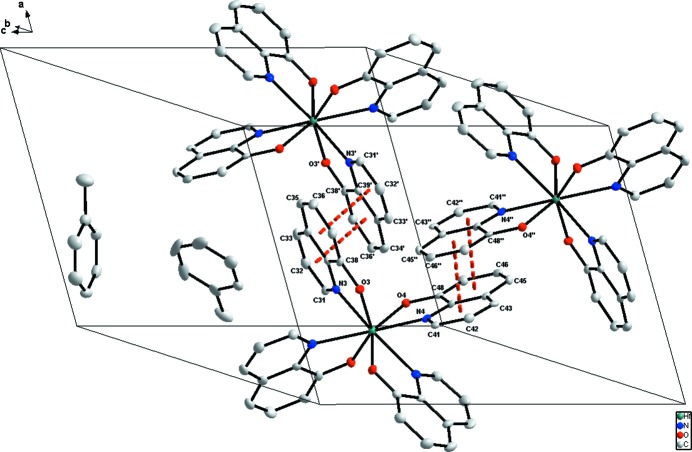
Graphical illustration of π-π interaction and stacking between different quinoline ligands of neighbouring molecules to produce a three dimensional network (displacement ellipsoids at the 50% probability level, H atoms omitted for clarity). Symmetry codes: (') 1 - x, -y, 1 - z; (") 1 - x, 1 - y, 1 - z.

## supplementary crystallographic information

### Comment

This study forms part of an ongoing researh project that investigates the chelating behaviour of *O*,*O*'- and *O*,*N*-bidentate ligands with hafnium(IV) and zirconium(IV) for possible separation of these two metals (Viljoen *et al.* 2008, 2009). The total separation of zircon ore(ZrSiO_4_), which contains traces of hafnium, is of most importance for nucleur aplications. A few hafnium complexes containing *β*-diketonato ligands have been reported by others (Zherikova *et al.* (2008); Demakopoulos *et al.* (1995)). An analogous zirconium complex has been reported by Lewis & Fay (1974).

The title compound [Hf(C_9_H_6_NO)_4_].2(C_7_H_8_), where C_9_H_6_NO ( Ox^-^) = 8-hydroxyquinoline and C_7_H_8 _= toluene, crystallizes in the form of yellow plate-like crystals of in the triclinic system (*P*1*, Z*=2) (Figure 1) with two toluene solvent molecules in the asymmetric unit. The Hf^IV^ atom is eight coordinated and surrounded by four chelating *β*-diketonato Ox^-^ ligands to give a square antiprismatic coordination polyhedron with a slight distortion towards a dodecahedral geometry. The Hf—O and Hf—N bond lengths vary from 2.085 (3) Å to 2.103 (3) Å and 2.391 (3) Å to 2.404 (3) Å, respectivily, and the O—Hf—N bite angles vary from 70.73 (11)° to 71.16 (11)° . The dihedral angle between the two phenyl rings in the quinoline ligands are all less than 1°, indicating a negligible distortion due to coordination or packing. The molecular units are connected by π-π interactions between different quinoline ligands of neighbouring molecules to produce a three dimensional network, with interplaner distances varying between 3.138 (1) Å and 3.208 (2) Å and centroid-to-centroid distances from 3.576 (1) Å and 4.074 (1) Å (*see* Figure 2). Lastly, a strong C—H···O hydrogen bonding interaction is observed between a solvent molecule and one of the oxygen atoms from a neighbouring metallic molecular group (*see* Table 1).

### Experimental

Chemicals were purchased from Sigma and Aldrich and used as received except for toluene which was dried by passage over alumina. Syntheses were perfomed using modified Schlenk conditions. OxH (0.369 g, 254 mmol) was added to a suspension of HfCl_4 _(0.201 g, 0.63 mmol) in toluene (10 ml). The dissolution turned into a slightly yellow solution after 10 min, and after refluxing for *ca* 20 h, the crude product was filtered and washed with toluene. The filtrate was slowly recrystallized at 253 K at near qauntitative yield. Spectroscopy data: ^1^H NMR (Benzene-*d_6_*;):*δ* = 6.70 (d, 1H, *J *= 6 Hz), 7.29 (dd, 2H, *J* = 7.8 Hz, 6 Hz), 7.36 (t, 2H, *J* = 7.8 Hz), 8.13 (d, 1H, *J* = 7.2 Hz); IR (ATR): *ν*(CO) 1659 cm^-1^.

### Refinement

The aromatic, methine, and methyl H atoms were placed in geometrically idealized positions (C—H = 0.93–0.98) and constrained to ride on their parent atoms with *U*_iso_(H) = 1.2*U*_eq_(C) for aromatic and methine, and *U*_iso_(H) = 1.5*U*_eq_(C) for methyl protons. Torsion angles for methyl protons were refined from electron density. The highest residual electron density was located 2.34 Å from H311 and was essentially meaningless.

### Crystal data

**Table:**

[Hf(C_9_H_6_NO)_4_]·2C_7_H_8_	*Z* = 2
*M**_r_* = 939.35	*F*(000) = 944
Triclinic, *P*1	*D*_x_ = 1.602 Mg m^−^^3^
Hall symbol: -P 1	Mo *K*α radiation, λ = 0.71073 Å
*a* = 11.3323 (5) Å	Cell parameters from 9377 reflections
*b* = 12.5539 (5) Å	θ = 2.6–28.1°
*c* = 15.7126 (7) Å	µ = 2.73 mm^−^^1^
α = 69.746 (2)°	*T* = 100 K
β = 69.700 (2)°	Plate, yellow
γ = 75.787 (2)°	0.22 × 0.10 × 0.04 mm
*V* = 1946.79 (14) Å^3^	

### Data collection

**Table:**

Bruker X8 APEXII 4K Kappa CCD diffractometer	8458 independent reflections
Radiation source: fine-focus sealed tube	7551 reflections with *I* > 2σ(*I*)
graphite	*R*_int_ = 0.044
ω and φ scans	θ_max_ = 27°, θ_min_ = 1.4°
Absorption correction: multi-scan (*SADABS*; Bruker, 2004)	*h* = −14→14
*T*_min_ = 0.585, *T*_max_ = 0.899	*k* = −16→16
22928 measured reflections	*l* = −20→20

### Refinement

**Table:**

Refinement on *F*^2^	Primary atom site location: structure-invariant direct methods
Least-squares matrix: full	Secondary atom site location: difference Fourier map
*R*[*F*^2^ > 2σ(*F*^2^)] = 0.033	Hydrogen site location: inferred from neighbouring sites
*wR*(*F*^2^) = 0.100	H-atom parameters constrained
*S* = 1.04	*w* = 1/[σ^2^(*F*_o_^2^) + (0.0618*P*)^2^] where *P* = (*F*_o_^2^ + 2*F*_c_^2^)/3
8458 reflections	(Δ/σ)_max_ = 0.002
534 parameters	Δρ_max_ = 1.16 e Å^−^^3^
0 restraints	Δρ_min_ = −0.81 e Å^−^^3^

### Special details

**Table:**

Geometry. All s.u.'s (except the s.u. in the dihedral angle between two l.s. planes) are estimated using the full covariance matrix. The cell s.u.'s are taken into account individually in the estimation of s.u.'s in distances, angles and torsion angles; correlations between s.u.'s in cell parameters are only used when they are defined by crystal symmetry. An approximate (isotropic) treatment of cell s.u.'s is used for estimating s.u.'s involving l.s. planes.
Refinement. Refinement of *F*^2^ against ALL reflections. The weighted *R*-factor *wR* and goodness of fit *S* are based on *F*^2^, conventional *R*-factors *R* are based on *F*, with *F* set to zero for negative *F*^2^. The threshold expression of *F*^2^ > 2σ(*F*^2^) is used only for calculating *R*-factors(gt) *etc*. and is not relevant to the choice of reflections for refinement. *R*-factors based on *F*^2^ are statistically about twice as large as those based on *F*, and *R*- factors based on ALL data will be even larger.

### Fractional atomic coordinates and isotropic or equivalent isotropic displacement parameters (Å^2^)

**Table:**

	*x*	*y*	*z*	*U*_iso_*/*U*_eq_	
C11	0.0301 (4)	0.2510 (4)	0.5067 (3)	0.0187 (9)	
H11	0.0671	0.3158	0.4665	0.022*	
C12	−0.0455 (4)	0.2069 (4)	0.4758 (3)	0.0221 (10)	
H12	−0.057	0.2415	0.4159	0.026*	
C13	−0.1014 (5)	0.1134 (4)	0.5345 (3)	0.0237 (10)	
H13	−0.1498	0.0829	0.5139	0.028*	
C14	−0.0872 (4)	0.0617 (4)	0.6263 (3)	0.0184 (9)	
C15	−0.1430 (5)	−0.0332 (4)	0.6941 (4)	0.0244 (10)	
H15	−0.195	−0.0678	0.6801	0.029*	
C16	−0.1200 (5)	−0.0747 (4)	0.7813 (3)	0.0239 (10)	
H16	−0.1573	−0.1376	0.8256	0.029*	
C17	−0.0420 (4)	−0.0252 (4)	0.8055 (3)	0.0200 (9)	
H17	−0.0293	−0.0549	0.8652	0.024*	
C18	0.0159 (4)	0.0675 (3)	0.7407 (3)	0.0151 (8)	
C19	−0.0091 (4)	0.1124 (3)	0.6511 (3)	0.0125 (8)	
C21	0.1816 (4)	0.2455 (3)	0.8712 (3)	0.0158 (9)	
H21	0.2533	0.1901	0.8658	0.019*	
C22	0.1269 (5)	0.2752 (4)	0.9567 (3)	0.0202 (9)	
H22	0.1611	0.2386	1.0072	0.024*	
C23	0.0237 (5)	0.3580 (4)	0.9650 (3)	0.0205 (10)	
H23	−0.0115	0.3791	1.0209	0.025*	
C24	−0.0297 (4)	0.4116 (4)	0.8891 (3)	0.0168 (9)	
C25	−0.1373 (5)	0.4969 (4)	0.8906 (3)	0.0219 (10)	
H25	−0.1785	0.5219	0.9443	0.026*	
C26	−0.1805 (5)	0.5423 (4)	0.8124 (3)	0.0249 (10)	
H26	−0.2515	0.5984	0.814	0.03*	
C27	−0.1215 (4)	0.5072 (3)	0.7294 (3)	0.0177 (9)	
H27	−0.1534	0.5403	0.6776	0.021*	
C28	−0.0158 (4)	0.4235 (3)	0.7253 (3)	0.0151 (9)	
C29	0.0305 (4)	0.3765 (3)	0.8060 (3)	0.0139 (8)	
C31	0.2648 (4)	0.5148 (3)	0.5866 (3)	0.0148 (8)	
H31	0.1885	0.5379	0.5712	0.018*	
C32	0.3397 (5)	0.5985 (4)	0.5694 (3)	0.0191 (9)	
H32	0.3126	0.6756	0.5433	0.023*	
C33	0.4522 (5)	0.5659 (4)	0.5911 (3)	0.0203 (10)	
H33	0.5025	0.6207	0.5796	0.024*	
C34	0.4924 (4)	0.4487 (4)	0.6312 (3)	0.0157 (9)	
C35	0.6077 (4)	0.4046 (4)	0.6546 (3)	0.0205 (9)	
H35	0.6635	0.454	0.6446	0.025*	
C36	0.6377 (5)	0.2882 (4)	0.6923 (3)	0.0224 (10)	
H36	0.7137	0.2598	0.7079	0.027*	
C37	0.5553 (4)	0.2116 (4)	0.7076 (3)	0.0199 (9)	
H37	0.5773	0.1336	0.734	0.024*	
C38	0.4435 (4)	0.2497 (3)	0.6844 (3)	0.0147 (8)	
C39	0.4106 (4)	0.3707 (3)	0.6462 (3)	0.0137 (8)	
C41	0.2922 (4)	−0.0275 (3)	0.6404 (3)	0.0124 (8)	
H41	0.2627	−0.0479	0.7062	0.015*	
C42	0.3422 (4)	−0.1154 (3)	0.5959 (3)	0.0165 (9)	
H42	0.3453	−0.1919	0.6319	0.02*	
C43	0.3857 (4)	−0.0878 (3)	0.4999 (3)	0.0152 (9)	
H43	0.4185	−0.1453	0.4698	0.018*	
C44	0.3809 (4)	0.0293 (3)	0.4459 (3)	0.0136 (8)	
C45	0.4271 (4)	0.0700 (3)	0.3460 (3)	0.0158 (9)	
H45	0.4624	0.0183	0.3103	0.019*	
C46	0.4198 (4)	0.1852 (4)	0.3018 (3)	0.0163 (9)	
H46	0.4487	0.2105	0.2361	0.02*	
C47	0.3701 (4)	0.2663 (3)	0.3526 (3)	0.0159 (9)	
H47	0.3674	0.3441	0.3202	0.019*	
C48	0.3249 (4)	0.2319 (3)	0.4504 (3)	0.0121 (8)	
C49	0.3295 (4)	0.1114 (3)	0.4969 (3)	0.0115 (8)	
C101	0.4169 (5)	0.8733 (4)	0.9727 (4)	0.0242 (10)	
C102	0.3554 (5)	0.8060 (4)	1.0597 (3)	0.0287 (11)	
H102	0.4019	0.7609	1.1014	0.034*	
C103	0.2263 (5)	0.8043 (4)	1.0863 (4)	0.0286 (11)	
H103	0.1871	0.7571	1.1449	0.034*	
C104	0.1550 (5)	0.8725 (4)	1.0261 (3)	0.0233 (10)	
H104	0.0679	0.8716	1.0438	0.028*	
C105	0.2150 (5)	0.9418 (4)	0.9396 (3)	0.0233 (10)	
H105	0.1678	0.9886	0.8988	0.028*	
C106	0.3449 (5)	0.9423 (4)	0.9129 (3)	0.0197 (9)	
H106	0.3843	0.9895	0.8542	0.024*	
C107	0.5587 (5)	0.8748 (5)	0.9431 (4)	0.0414 (14)	
H10A	0.5795	0.8976	0.9879	0.062*	
H10B	0.5836	0.9283	0.8814	0.062*	
H10C	0.6029	0.7996	0.9411	0.062*	
C201	0.2346 (5)	0.6255 (4)	0.8215 (3)	0.0232 (10)	
C202	0.2835 (4)	0.5123 (4)	0.8235 (3)	0.0206 (9)	
H202	0.248	0.474	0.7994	0.025*	
C203	0.3829 (5)	0.4553 (4)	0.8601 (3)	0.0281 (11)	
H203	0.4145	0.3796	0.8599	0.034*	
C204	0.4356 (6)	0.5098 (5)	0.8971 (4)	0.0394 (14)	
H204	0.5034	0.4712	0.9215	0.047*	
C205	0.3890 (7)	0.6201 (5)	0.8981 (4)	0.0459 (17)	
H205	0.4239	0.6565	0.9242	0.055*	
C206	0.2888 (6)	0.6785 (4)	0.8601 (3)	0.0389 (15)	
H206	0.2578	0.7542	0.8607	0.047*	
C207	0.1292 (5)	0.6861 (4)	0.7794 (4)	0.0353 (13)	
H20A	0.1627	0.7132	0.7117	0.053*	
H20B	0.0867	0.7501	0.8039	0.053*	
H20C	0.0699	0.6344	0.7951	0.053*	
N1	0.0504 (3)	0.2037 (3)	0.5909 (2)	0.0148 (7)	
N2	0.1342 (3)	0.2939 (3)	0.7981 (2)	0.0140 (7)	
N3	0.2993 (3)	0.4043 (3)	0.6239 (2)	0.0141 (7)	
N4	0.2853 (3)	0.0823 (3)	0.5933 (2)	0.0130 (7)	
O1	0.0910 (3)	0.1197 (2)	0.75695 (19)	0.0144 (6)	
O2	0.0440 (3)	0.3817 (2)	0.6519 (2)	0.0141 (6)	
O3	0.3619 (3)	0.1828 (2)	0.6929 (2)	0.0142 (6)	
O4	0.2786 (3)	0.3022 (2)	0.5050 (2)	0.0134 (6)	
Hf	0.194221 (16)	0.246881 (12)	0.651652 (11)	0.01143 (7)	

### Atomic displacement parameters (Å^2^)

**Table:**

	*U*^11^	*U*^22^	*U*^33^	*U*^12^	*U*^13^	*U*^23^
C11	0.016 (2)	0.022 (2)	0.018 (2)	−0.0016 (18)	−0.0059 (18)	−0.0054 (17)
C12	0.018 (3)	0.032 (2)	0.017 (2)	0.000 (2)	−0.0085 (19)	−0.0083 (19)
C13	0.013 (2)	0.031 (2)	0.035 (3)	0.0034 (19)	−0.013 (2)	−0.016 (2)
C14	0.013 (2)	0.019 (2)	0.027 (2)	−0.0022 (17)	−0.0079 (19)	−0.0091 (18)
C15	0.017 (3)	0.024 (2)	0.038 (3)	−0.0043 (19)	−0.010 (2)	−0.012 (2)
C16	0.020 (3)	0.023 (2)	0.028 (3)	−0.0068 (19)	−0.005 (2)	−0.0058 (19)
C17	0.018 (2)	0.022 (2)	0.018 (2)	−0.0031 (18)	−0.0045 (19)	−0.0058 (18)
C18	0.012 (2)	0.0166 (19)	0.016 (2)	−0.0025 (17)	−0.0006 (17)	−0.0075 (16)
C19	0.008 (2)	0.0133 (18)	0.016 (2)	−0.0005 (16)	−0.0033 (17)	−0.0050 (16)
C21	0.015 (2)	0.0147 (19)	0.018 (2)	−0.0002 (16)	−0.0058 (18)	−0.0057 (16)
C22	0.023 (3)	0.024 (2)	0.015 (2)	−0.0067 (19)	−0.0075 (19)	−0.0038 (18)
C23	0.021 (3)	0.026 (2)	0.014 (2)	−0.0077 (19)	0.0015 (19)	−0.0096 (18)
C24	0.016 (2)	0.020 (2)	0.014 (2)	−0.0086 (18)	0.0002 (18)	−0.0039 (17)
C25	0.022 (3)	0.024 (2)	0.019 (2)	−0.0049 (19)	0.0013 (19)	−0.0109 (18)
C26	0.019 (3)	0.024 (2)	0.031 (3)	0.0039 (19)	−0.005 (2)	−0.013 (2)
C27	0.013 (2)	0.019 (2)	0.018 (2)	−0.0046 (17)	−0.0010 (18)	−0.0038 (17)
C28	0.019 (2)	0.0137 (18)	0.014 (2)	−0.0060 (17)	−0.0025 (18)	−0.0040 (16)
C29	0.012 (2)	0.0146 (19)	0.016 (2)	−0.0055 (16)	−0.0013 (17)	−0.0053 (16)
C31	0.015 (2)	0.0174 (19)	0.015 (2)	−0.0035 (17)	−0.0039 (17)	−0.0075 (16)
C32	0.024 (3)	0.016 (2)	0.016 (2)	−0.0032 (18)	−0.0039 (19)	−0.0054 (17)
C33	0.026 (3)	0.021 (2)	0.017 (2)	−0.0075 (19)	−0.0012 (19)	−0.0105 (18)
C34	0.015 (2)	0.021 (2)	0.013 (2)	−0.0083 (17)	0.0011 (17)	−0.0097 (17)
C35	0.017 (2)	0.029 (2)	0.021 (2)	−0.0114 (19)	−0.0008 (19)	−0.0129 (19)
C36	0.015 (2)	0.032 (2)	0.029 (3)	−0.0004 (19)	−0.008 (2)	−0.018 (2)
C37	0.019 (2)	0.023 (2)	0.022 (2)	−0.0014 (19)	−0.0065 (19)	−0.0122 (18)
C38	0.017 (2)	0.0164 (19)	0.015 (2)	−0.0048 (17)	−0.0049 (18)	−0.0077 (16)
C39	0.011 (2)	0.020 (2)	0.013 (2)	−0.0042 (17)	0.0010 (17)	−0.0102 (16)
C41	0.009 (2)	0.0159 (18)	0.014 (2)	−0.0028 (16)	−0.0040 (16)	−0.0046 (16)
C42	0.015 (2)	0.0141 (19)	0.022 (2)	−0.0016 (17)	−0.0059 (18)	−0.0061 (17)
C43	0.007 (2)	0.0175 (19)	0.024 (2)	−0.0026 (16)	−0.0027 (17)	−0.0105 (17)
C44	0.011 (2)	0.0152 (19)	0.019 (2)	−0.0030 (16)	−0.0063 (17)	−0.0071 (16)
C45	0.014 (2)	0.020 (2)	0.018 (2)	−0.0010 (17)	−0.0032 (18)	−0.0129 (17)
C46	0.012 (2)	0.026 (2)	0.014 (2)	−0.0055 (18)	−0.0057 (17)	−0.0061 (17)
C47	0.016 (2)	0.0165 (19)	0.017 (2)	0.0002 (17)	−0.0076 (18)	−0.0048 (16)
C48	0.008 (2)	0.0155 (19)	0.016 (2)	−0.0054 (16)	−0.0030 (16)	−0.0052 (16)
C49	0.013 (2)	0.0126 (18)	0.0120 (19)	−0.0058 (16)	−0.0041 (16)	−0.0036 (15)
C101	0.022 (3)	0.024 (2)	0.029 (3)	−0.002 (2)	−0.008 (2)	−0.012 (2)
C102	0.031 (3)	0.028 (2)	0.021 (3)	0.002 (2)	−0.012 (2)	−0.002 (2)
C103	0.032 (3)	0.027 (2)	0.021 (2)	−0.006 (2)	−0.005 (2)	−0.0017 (19)
C104	0.016 (2)	0.023 (2)	0.032 (3)	−0.0052 (19)	−0.003 (2)	−0.012 (2)
C105	0.029 (3)	0.018 (2)	0.029 (3)	0.0045 (19)	−0.018 (2)	−0.0111 (19)
C106	0.023 (3)	0.017 (2)	0.019 (2)	−0.0089 (18)	−0.0017 (19)	−0.0051 (17)
C107	0.024 (3)	0.051 (3)	0.045 (4)	−0.005 (3)	−0.005 (3)	−0.014 (3)
C201	0.027 (3)	0.021 (2)	0.013 (2)	−0.012 (2)	0.0096 (19)	−0.0033 (17)
C202	0.017 (2)	0.027 (2)	0.019 (2)	−0.0059 (19)	−0.0004 (19)	−0.0112 (18)
C203	0.028 (3)	0.036 (3)	0.018 (2)	−0.009 (2)	−0.003 (2)	−0.006 (2)
C204	0.034 (3)	0.069 (4)	0.017 (3)	−0.027 (3)	−0.004 (2)	−0.006 (3)
C205	0.071 (5)	0.063 (4)	0.020 (3)	−0.052 (4)	−0.005 (3)	−0.009 (3)
C206	0.068 (5)	0.025 (2)	0.020 (3)	−0.026 (3)	0.008 (3)	−0.009 (2)
C207	0.029 (3)	0.027 (3)	0.027 (3)	0.005 (2)	0.003 (2)	0.001 (2)
N1	0.0113 (19)	0.0180 (17)	0.0155 (18)	0.0010 (14)	−0.0043 (15)	−0.0068 (14)
N2	0.0152 (19)	0.0123 (15)	0.0143 (18)	−0.0064 (14)	−0.0028 (15)	−0.0021 (13)
N3	0.0129 (19)	0.0173 (17)	0.0130 (17)	−0.0036 (14)	−0.0005 (15)	−0.0076 (14)
N4	0.0104 (18)	0.0131 (16)	0.0185 (18)	−0.0021 (14)	−0.0061 (15)	−0.0061 (14)
O1	0.0159 (16)	0.0165 (13)	0.0131 (14)	−0.0052 (12)	−0.0043 (12)	−0.0049 (11)
O2	0.0149 (16)	0.0143 (13)	0.0139 (15)	0.0001 (12)	−0.0047 (12)	−0.0059 (11)
O3	0.0119 (16)	0.0149 (13)	0.0177 (15)	−0.0019 (11)	−0.0042 (12)	−0.0071 (11)
O4	0.0143 (16)	0.0118 (13)	0.0149 (15)	−0.0054 (11)	−0.0014 (12)	−0.0050 (11)
Hf	0.01205 (11)	0.01173 (10)	0.01191 (10)	−0.00236 (7)	−0.00353 (7)	−0.00454 (7)

### Geometric parameters (Å, °)

**Table:**

C11—N1	1.325 (5)	C41—C42	1.408 (5)
C11—C12	1.409 (6)	C41—H41	0.93
C11—H11	0.93	C42—C43	1.357 (6)
C12—C13	1.356 (7)	C42—H42	0.93
C12—H12	0.93	C43—C44	1.419 (5)
C13—C14	1.411 (6)	C43—H43	0.93
C13—H13	0.93	C44—C49	1.413 (5)
C14—C15	1.407 (6)	C44—C45	1.417 (6)
C14—C19	1.423 (6)	C45—C46	1.366 (6)
C15—C16	1.380 (7)	C45—H45	0.93
C15—H15	0.93	C46—C47	1.400 (6)
C16—C17	1.407 (6)	C46—H46	0.93
C16—H16	0.93	C47—C48	1.383 (6)
C17—C18	1.382 (6)	C47—H47	0.93
C17—H17	0.93	C48—O4	1.332 (5)
C18—O1	1.326 (5)	C48—C49	1.432 (5)
C18—C19	1.424 (6)	C49—N4	1.363 (5)
C19—N1	1.360 (5)	C101—C102	1.377 (7)
C21—N2	1.328 (5)	C101—C106	1.386 (6)
C21—C22	1.409 (6)	C101—C107	1.513 (7)
C21—H21	0.93	C102—C103	1.378 (7)
C22—C23	1.362 (6)	C102—H102	0.93
C22—H22	0.93	C103—C104	1.382 (7)
C23—C24	1.411 (6)	C103—H103	0.93
C23—H23	0.93	C104—C105	1.377 (7)
C24—C25	1.410 (6)	C104—H104	0.93
C24—C29	1.413 (6)	C105—C106	1.385 (7)
C25—C26	1.367 (6)	C105—H105	0.93
C25—H25	0.93	C106—H106	0.93
C26—C27	1.410 (6)	C107—H10A	0.96
C26—H26	0.93	C107—H10B	0.96
C27—C28	1.385 (6)	C107—H10C	0.96
C27—H27	0.93	C201—C202	1.386 (6)
C28—O2	1.329 (5)	C201—C206	1.389 (7)
C28—C29	1.422 (6)	C201—C207	1.482 (7)
C29—N2	1.365 (5)	C202—C203	1.371 (7)
C31—N3	1.322 (5)	C202—H202	0.93
C31—C32	1.407 (6)	C203—C204	1.371 (7)
C31—H31	0.93	C203—H203	0.93
C32—C33	1.364 (6)	C204—C205	1.359 (9)
C32—H32	0.93	C204—H204	0.93
C33—C34	1.414 (6)	C205—C206	1.393 (9)
C33—H33	0.93	C205—H205	0.93
C34—C35	1.409 (6)	C206—H206	0.93
C34—C39	1.418 (6)	C207—H20A	0.96
C35—C36	1.377 (6)	C207—H20B	0.96
C35—H35	0.93	C207—H20C	0.96
C36—C37	1.407 (6)	N1—Hf	2.395 (3)
C36—H36	0.93	N2—Hf	2.400 (3)
C37—C38	1.367 (6)	N3—Hf	2.391 (3)
C37—H37	0.93	N4—Hf	2.404 (3)
C38—O3	1.340 (5)	O1—Hf	2.098 (3)
C38—C39	1.433 (6)	O2—Hf	2.085 (3)
C39—N3	1.353 (5)	O3—Hf	2.103 (3)
C41—N4	1.317 (5)	O4—Hf	2.096 (3)
			
N1—C11—C12	122.4 (4)	C48—C47—H47	119.7
N1—C11—H11	118.8	C46—C47—H47	119.7
C12—C11—H11	118.8	O4—C48—C47	125.2 (4)
C13—C12—C11	119.2 (4)	O4—C48—C49	117.2 (3)
C13—C12—H12	120.4	C47—C48—C49	117.7 (4)
C11—C12—H12	120.4	N4—C49—C44	123.0 (4)
C12—C13—C14	121.0 (4)	N4—C49—C48	115.2 (3)
C12—C13—H13	119.5	C44—C49—C48	121.8 (4)
C14—C13—H13	119.5	C102—C101—C106	118.2 (5)
C15—C14—C13	126.1 (4)	C102—C101—C107	121.5 (5)
C15—C14—C19	118.2 (4)	C106—C101—C107	120.2 (5)
C13—C14—C19	115.7 (4)	C101—C102—C103	121.4 (5)
C16—C15—C14	119.6 (4)	C101—C102—H102	119.3
C16—C15—H15	120.2	C103—C102—H102	119.3
C14—C15—H15	120.2	C102—C103—C104	120.2 (5)
C15—C16—C17	122.2 (4)	C102—C103—H103	119.9
C15—C16—H16	118.9	C104—C103—H103	119.9
C17—C16—H16	118.9	C105—C104—C103	119.0 (5)
C18—C17—C16	120.1 (4)	C105—C104—H104	120.5
C18—C17—H17	119.9	C103—C104—H104	120.5
C16—C17—H17	119.9	C104—C105—C106	120.6 (4)
O1—C18—C17	124.5 (4)	C104—C105—H105	119.7
O1—C18—C19	117.4 (4)	C106—C105—H105	119.7
C17—C18—C19	118.1 (4)	C105—C106—C101	120.6 (4)
N1—C19—C14	123.1 (4)	C105—C106—H106	119.7
N1—C19—C18	115.1 (4)	C101—C106—H106	119.7
C14—C19—C18	121.7 (4)	C101—C107—H10A	109.5
N2—C21—C22	122.1 (4)	C101—C107—H10B	109.5
N2—C21—H21	118.9	H10A—C107—H10B	109.5
C22—C21—H21	118.9	C101—C107—H10C	109.5
C23—C22—C21	119.7 (4)	H10A—C107—H10C	109.5
C23—C22—H22	120.1	H10B—C107—H10C	109.5
C21—C22—H22	120.1	C202—C201—C206	117.1 (5)
C22—C23—C24	120.0 (4)	C202—C201—C207	120.8 (4)
C22—C23—H23	120	C206—C201—C207	122.1 (5)
C24—C23—H23	120	C203—C202—C201	121.8 (4)
C25—C24—C23	124.7 (4)	C203—C202—H202	119.1
C25—C24—C29	118.6 (4)	C201—C202—H202	119.1
C23—C24—C29	116.7 (4)	C204—C203—C202	120.0 (5)
C26—C25—C24	119.3 (4)	C204—C203—H203	120
C26—C25—H25	120.4	C202—C203—H203	120
C24—C25—H25	120.4	C205—C204—C203	120.1 (6)
C25—C26—C27	122.5 (4)	C205—C204—H204	120
C25—C26—H26	118.7	C203—C204—H204	120
C27—C26—H26	118.7	C204—C205—C206	120.0 (5)
C28—C27—C26	119.9 (4)	C204—C205—H205	120
C28—C27—H27	120	C206—C205—H205	120
C26—C27—H27	120	C201—C206—C205	121.0 (5)
O2—C28—C27	124.6 (4)	C201—C206—H206	119.5
O2—C28—C29	117.3 (4)	C205—C206—H206	119.5
C27—C28—C29	118.0 (4)	C201—C207—H20A	109.5
N2—C29—C24	122.8 (4)	C201—C207—H20B	109.5
N2—C29—C28	115.4 (4)	H20A—C207—H20B	109.5
C24—C29—C28	121.7 (4)	C201—C207—H20C	109.5
N3—C31—C32	122.3 (4)	H20A—C207—H20C	109.5
N3—C31—H31	118.9	H20B—C207—H20C	109.5
C32—C31—H31	118.9	C11—N1—C19	118.5 (4)
C33—C32—C31	119.6 (4)	C11—N1—Hf	128.7 (3)
C33—C32—H32	120.2	C19—N1—Hf	112.7 (3)
C31—C32—H32	120.2	C21—N2—C29	118.5 (4)
C32—C33—C34	119.9 (4)	C21—N2—Hf	129.1 (3)
C32—C33—H33	120	C29—N2—Hf	112.2 (3)
C34—C33—H33	120	C31—N3—C39	118.9 (4)
C35—C34—C33	125.0 (4)	C31—N3—Hf	128.1 (3)
C35—C34—C39	118.5 (4)	C39—N3—Hf	112.9 (2)
C33—C34—C39	116.4 (4)	C41—N4—C49	118.0 (3)
C36—C35—C34	119.9 (4)	C41—N4—Hf	129.4 (3)
C36—C35—H35	120	C49—N4—Hf	112.5 (2)
C34—C35—H35	120	C18—O1—Hf	123.2 (3)
C35—C36—C37	121.2 (4)	C28—O2—Hf	123.8 (3)
C35—C36—H36	119.4	C38—O3—Hf	123.4 (2)
C37—C36—H36	119.4	C48—O4—Hf	123.6 (2)
C38—C37—C36	121.3 (4)	O2—Hf—O4	94.50 (11)
C38—C37—H37	119.4	O2—Hf—O1	97.00 (11)
C36—C37—H37	119.4	O4—Hf—O1	141.84 (10)
O3—C38—C37	125.4 (4)	O2—Hf—O3	142.31 (11)
O3—C38—C39	116.5 (4)	O4—Hf—O3	97.89 (11)
C37—C38—C39	118.1 (4)	O1—Hf—O3	94.85 (11)
N3—C39—C34	122.9 (4)	O2—Hf—N3	78.53 (12)
N3—C39—C38	116.0 (4)	O4—Hf—N3	73.74 (11)
C34—C39—C38	121.0 (4)	O1—Hf—N3	144.27 (11)
N4—C41—C42	123.1 (4)	O3—Hf—N3	71.16 (11)
N4—C41—H41	118.4	O2—Hf—N1	73.68 (11)
C42—C41—H41	118.4	O4—Hf—N1	77.94 (11)
C43—C42—C41	119.6 (4)	O1—Hf—N1	70.73 (11)
C43—C42—H42	120.2	O3—Hf—N1	143.78 (11)
C41—C42—H42	120.2	N3—Hf—N1	138.19 (12)
C42—C43—C44	119.5 (4)	O2—Hf—N2	71.04 (11)
C42—C43—H43	120.2	O4—Hf—N2	145.17 (11)
C44—C43—H43	120.2	O1—Hf—N2	72.67 (11)
C49—C44—C45	117.8 (4)	O3—Hf—N2	78.70 (11)
C49—C44—C43	116.8 (4)	N3—Hf—N2	72.39 (11)
C45—C44—C43	125.4 (4)	N1—Hf—N2	124.51 (12)
C46—C45—C44	120.1 (4)	O2—Hf—N4	142.23 (11)
C46—C45—H45	119.9	O4—Hf—N4	70.84 (11)
C44—C45—H45	119.9	O1—Hf—N4	77.98 (11)
C45—C46—C47	122.0 (4)	O3—Hf—N4	75.31 (11)
C45—C46—H46	119	N3—Hf—N4	126.37 (12)
C47—C46—H46	119	N1—Hf—N4	69.32 (11)
C48—C47—C46	120.6 (4)	N2—Hf—N4	138.57 (11)

### Hydrogen-bond geometry (Å, °)

**Table:**

*D*—H···*A*	*D*—H	H···*A*	*D*···*A*	*D*—H···*A*
C105—H105···O1^i^	0.93	2.56	3.467 (5)	166

Symmetry codes: (i) *x*, *y*+1, *z*.
